# Racial/Ethnic Disparities in Dietary Intake of U.S. Children Participating in WIC

**DOI:** 10.3390/nu11112607

**Published:** 2019-10-31

**Authors:** Meghan C. Zimmer, Veronica Rubio, Kristina W. Kintziger, Cristina Barroso

**Affiliations:** 1 Department of Public Health, University of Tennessee, Knoxville, TN 37996, USA,; 2 Department of Nutrition, University of Tennessee, Knoxville, TN 37996, USA,

**Keywords:** WIC, NHANES, children, disparities, low-income, nutrient intake, dietary intake

## Abstract

Recent studies have assessed diet quality of low-income U.S. children participating in the Special Supplemental Nutrition Program for Women, Infants, and Children (WIC), but differences by race/ethnicity remain unknown. We assessed racial/ethnic disparities in nutrient intake from dietary sources (not supplements) among children participating in WIC, with a focus on priority nutrients and food groups for future WIC food package revisions, as described in a recent report by the National Academies of Sciences, Engineering, and Medicine (NASEM). We used data from the 2011–2014 National Health and Nutrition Examination Surveys (NHANES) and multivariable linear regression analysis to evaluate relationships between race/ethnicity and nutrient/food group intake of children participating in WIC. All data were analyzed using SAS 9.4 survey procedures, accounting for the complex survey design of the NHANES. Compared to non-Hispanic White children, Hispanic children had diets with better nutrient distribution and lower dietary energy density, while non-Hispanic Black children had diets with poorer nutrient intake. Hispanic children had higher potassium and fiber intake, and consumed more legumes, while non-Hispanic Black children had lower calcium and vitamin D intake, higher sodium intake, and lower total dairy intake, compared to non-Hispanic White children. These findings can inform WIC nutrition education messages and future food package revisions.

## 1. Introduction

The Special Supplemental Nutrition Program for Women, Infants, and Children (WIC) is a United States nutrition assistance program that has significant capacity for early intervention to establish adequate nutritional intake among children and women in low-income households. WIC provides nutrient-dense foods, nutrition education, and referrals to health/social services to children and women at nutrition risk living in low-income (100%–185% of the federal poverty level) households [1]. WIC food groups include grains, fruits and vegetables, dairy and protein. Specific food items and quantities vary by the food package received. WIC food packages are available for pregnant women, breastfeeding and non-breastfeeding mothers, infants, and children 1-4 years of age. Children have always been the largest category of WIC participants [2]. Approximately half of all infants in the U.S., and one quarter of all children less than five years of age, participate in WIC [1]. Several studies published within the past year have used nationally representative data to assess diet quality of children participating in WIC [3,4,5,6,7,8], but differences by race/ethnicity remain unknown.

Racial/ethnic disparities in nutrient intake of children participating in WIC are important considerations for WIC nutrition education and future food package revisions. The National Academies of Sciences, Engineering, and Medicine (NASEM) recently reviewed the WIC food package and defined nutrient inadequacies and excesses linked to adverse health consequences relevant to WIC-participating populations, and determined potential food package changes [9]. However, it is unclear what impact future food package revisions will have by race/ethnicity. Studies pertaining to the previous comprehensive (2009) WIC food package revisions have shown the revisions to be associated with improvements in dietary intake of children and women [8], and different changes in child dietary intake by race/ethnicity [10,11]. However, trends by race/ethnicity have not been assessed using nationally representative data. We addressed those knowledge gaps by using nationally representative data from the National Health and Nutrition Examination Survey (NHANES) 2011–2014 to assess racial/ethnic disparities in nutrient and food group intake from dietary sources (not supplements) among children participating in WIC in a time period post-revisions. In addition, we focused on priority nutrients and food groups for future WIC food package revisions, as described in the NASEM report [9]. 

## 2. Materials and Methods 

### 2.1. Data Source

This national cross-sectional study was conducted using data from the National Health and Nutrition Examination Survey (NHANES). The NHANES is a continuous survey conducted by the National Center for Health Statistics (NCHS) using a complex, multistage probability cluster-sampling design that is representative of the U.S. civilian, noninstitutionalized population [12]. NHANES and its related nutritional component, *What We Eat In America*, are designed to monitor the health and nutritional status of non-institutionalized civilians in the U.S. [13]. NHANES participants complete physical examinations and comprehensive questionnaires at NHANES Mobile Examination Centers and in participants’ homes. 

The NHANES collected dietary data via 24-hour recall assessments using the United States Department of Agriculture (USDA) Automated Multiple Pass Method [14]. A parent or caretaker served as a proxy respondent for children younger than six years old. The NHANES collects two days of diet recall. The first recall is collected in-person at the mobile examination center, and the second is collected via phone 3–10 days later [14]. We used the first day of diet recall to promote comparability between our results and other studies in the literature that used NHANES data to assess dietary intake of children participating in WIC [7,8]. Results based on a single 24-hour recall are sufficient to estimate population means because the effects of random errors associated with dietary recall, including day-to-day variability, are generally assumed to cancel out if days of the week are evenly represented [14]. NHANES dietary data, and the associated dietary sample weights, are commonly used to describe food and nutrient intakes on a given day, and they are also useful for descriptive and analytical epidemiologic purposes [14]. The dietary data were processed using the USDA Food Patterns Equivalents Database, which converts 7000+ individually reported food and beverage items into 37 disaggregated USDA food patterns components (e.g., added sugars, saturated fat, etc.) [14], thus allowing for assessment of nutrient distribution across the diet. The NCHS Research Ethics Review Board reviewed and approved all study protocols for the NHANES. Since all NHANES data were de-identified and did not contain sensitive information, this study was exempt from further review at the university level. 

### 2.2. Sample

The sample included children 1–4 years of age from households income-eligible for WIC (100%–185% of the federal poverty level) with complete information on WIC participation status and one full day of dietary intake. Children who were breastfeeding or had unreliable, missing, or incomplete recall data were excluded from the sample. We defined WIC participation status using the household-level question due to larger sample size, and in order to account for potential spillover effects from siblings participating in WIC [15]. We assessed dietary intake for four racial/ethnic groups: non-Hispanic White (N-H White), non-Hispanic Black (N-H Black), Hispanic, and other/mixed. The Hispanic ethnic group included Mexican-American and other Hispanics. The other/mixed group included non-Hispanic Asian, mixed race, and other. We combined data from two cycles (2011–2012 and 2013–2014) of the NHANES to create a sample of the most current national dietary estimates that include a four-year sampling design that oversampled non-Hispanic Asians; oversampling of non-Hispanic Asians started in 2011 [16], information on WIC participation status was not available in the 2015–2016 NHANES [17], and dietary data are not yet available in the 2018-2019 NHANES.

### 2.3. Statistical Analysis

Distribution of socio-demographic characteristics (including age, sex, race/ethnicity, household income, household size, and total energy intake) was assessed by Wald Chi-squared test. The primary measure for this study was population mean nutrient/food group intake, reported as least-square means with standard error. The variables selected for analysis included nutrients linked to adverse health consequences relevant to WIC children, and priority food groups for future WIC food package revisions, as described in the NASEM report [9]. Analyses for nutrient intake were performed for children 1–4, and analyses for food group intake were for children 2–4, to promote comparability between our results and the age categories for priority nutrients/food groups in the NASEM report [9].

Multivariable linear regression was used to compare nutrient/food group intake of non-Hispanic Black, Hispanic, and other/mixed race children to a reference group of non-Hispanic White children. The model was adjusted for all relevant covariates, including age, sex, household income (using NHANES-provided poverty income ratio), and total energy intake. Analyses were weighted using the NHANES day-1 dietary sample weight to adjust for day of week for diet recall and dietary interview specific non-response [14]. All data were analyzed using SAS 9.4 survey procedures (SAS Institute, Cary, NC) with appropriate weighting and clustering to account for the differential probabilities of selection, non-response, and oversampling associated with the multistage, probability cluster-sampling design of the NHANES. For all statistical analyses, a *p*-value of <0.05 was considered statistically significant.

## 3. Results

Children 1-4 years of age who consumed breast milk (*n* = 15), had unreliable dietary recall status (*n* = 34), or missing/incomplete dietary data (*n* = 89) were excluded. Table 1 shows socio-demographic characteristics of the final sample. The sample comprised 1072 children 1–4 years of age that had complete information for one day of dietary intake in the NHANES 2011–2014. Of the 1072 children, 727 (67.8%) were living in households receiving WIC benefits and 345 (32.2%) were living in households that were income-eligible but not participating in the WIC program at the time of the NHANES interview. In the sample of children 2–4 years of age, there were 804 children with complete information for one day of dietary intake and WIC participation status that were included in the NHANES 2011–2014. Of the 804 children, 509 (63.3%) were living in households receiving WIC benefits and 295 (58.0%) were living in households that were income-eligible but not participating in the WIC program at the time of the NHANES interview. The final model, used to assess nutrient intake in the sample of children 1–4 and food group intake in the sample of children 2–4, was adjusted for all relevant covariates, including age, sex, household income, and total energy intake. 

In a nationally representative sample of U.S. children participating in the WIC program, racial/ethnic disparities in nutrient intake were observed. Compared to non-Hispanic White children, Hispanic children had diets with lower energy density and better nutrient distribution while non-Hispanic Black children had nutrient intake estimates that were poorer. Table 2 shows racial/ethnic differences in nutrient intake of children, with a focus on nutrient inadequacies and excesses linked to adverse health consequences relevant to children participating in WIC [9]. Among U.S. children participating in WIC, dietary energy density of Hispanic children (1.6 ± SE <0.1) was significantly lower (*p* < 0.001) than non-Hispanic White children (1.9 ± SE 0.1). Nutrient intake of Hispanic children was greater than non-Hispanic White children for fiber (12.1 g ± SE 0.5 vs. 10.7 g ± SE 0.3, *p* = 0.026), and potassium (2071 mg ± SE 44 vs. 1936 mg ± SE 63, *p* = 0.038). Conversely, non-Hispanic Black children had nutrient distribution across the diet that was poorer than their non-Hispanic White counterparts. Nutrient intake of non-Hispanic Black children was lower than that of non-Hispanic White children for calcium (826 mg ± SE 39 vs. 992 mg ± SE 50, *p* = 0.009) and vitamin D (5.7 mcg ± SE 0.4 vs. 7.5 mcg ± SE 0.6, *p* = 0.012), and higher in sodium (2136 mg ± SE 51 vs. 1906 mg ± SE 62, *p* = 0.006). There was one instance of better nutrient intake among non-Hispanic Black children compared to non-Hispanic White children; saturated fat intake was lower (17.5 g ± SE 0.5 vs. 20.2 g ± SE 1.1, *p* = 0.016). Mean estimates for nutrient intake on a given day for children of all race/ethnicities appeared to fall short of several dietary recommendations, including fiber, potassium, vitamin D, and exceeded recommendations for sodium, but met recommendations for calcium, iron, and zinc. Further research is needed to statistically compare intake by race/ethnicity to dietary guidelines. 

Racial/ethnic differences in food group intake of children participating in WIC were analyzed to determine differences in dietary intake that may contribute to the observed differences in nutrient intake. As shown in Table 3, non-Hispanic Black children consumed less dairy (1.75 c-eq ± SE 0.15) compared to non-Hispanic White children (2.33 c-eq ± SE 0.22, *p* = 0.020). Hispanic children consumed more legumes than non-Hispanic White children (0.16 c-eq ± SE 0.02 vs. 0.05 c-eq ± SE 0.03, *p* = 0.001). Total protein food consumption of non-Hispanic Black (3.61 oz-eq ± SE 0.29, *p* < 0.001) and Hispanic (2.92 oz-eq ± SE 0.15, *p* = 0.021) children exceeded that of non-Hispanic White children (2.19 oz-eq ± SE 0.23). Importantly, among WIC-participating children of all race/ethnicities, mean estimate of intake on a given day for several food groups appeared to fall short of weekly recommendations converted to a per-day basis, including seafood, total vegetables, whole grains, nuts and seeds, total dairy, and legumes, though further research is needed to statistically compare intake by race/ethnicity to dietary guidelines.

## 4. Discussion

The relationship between nutritional status and chronic disease is a major focus of public health nutrition research and practice. Low-income, minority populations are disproportionately impacted by obesity and chronic disease [19], and evidence continues to emerge demonstrating socio-economic gradients in diet quality [20]. The WIC program has significant potential to increase access to nutritious foods among women and children living in low-income households in the U.S., and reduce associated diet-related disparities. Despite the fact that many studies published within the past year have assessed diet quality of WIC participants using nationally representative data [3,4,5,6,7,8], none have specifically addressed racial/ethnic disparities in dietary intake of children participating in WIC. Our findings highlight differences in nutrient status of WIC-participating children across racial/ethnic groups, and suggest that Hispanic children have diets with better nutrient distribution and lower dietary energy density, while non-Hispanic Black children have diets with poorer nutrient intake, compared to non-Hispanic White children. Our findings are consistent with a previous study that directly compared diet quality of Hispanic and non-Hispanic Black WIC participants using regional data from Chicago, IL, USA prior to the comprehensive (2009) WIC food package revisions. The study found diets of Hispanic participants to have better nutrient distribution (high in vitamin A, calcium, dairy, whole gains, fruit) and be lower in energy-dense foods (lower percentage of calories from sweetened beverages, sodium, added sugars, fat), when compared to a non-Hispanic Black sample [11]. 

Calcium and vitamin D intake of non-Hispanic Black children 1–4 years old who were participating in WIC were significantly lower than their non-Hispanic White counterparts. For non-Hispanic Black children, average calcium intake met guidelines for children 1–3 years of age but fell short of the guidelines for 4–5 years of age. Children in all other racial/ethnic groups had average calcium intake that met or exceeded recommendations for children 1–5, though further research is needed to statistically compare intake by race/ethnicity to dietary guidelines. Understanding disparities in calcium and vitamin D intake is important because both are short-fall nutrients, identified in the Dietary Guidelines for Americans as being under-consumed by a significant portion of Americans [21]. In addition, a report by the NASEM identified calcium and vitamin D as nutrients linked to adverse health consequences that are relevant to WIC-participating populations [9]. 

In our analysis of food group intake for U.S. children participating in WIC, we found non-Hispanic Black children to consume less total dairy than their non-Hispanic White counterparts, which may contribute to the lower calcium and vitamin D intakes, given that three servings of fortified milk provide 70% of calcium and vitamin D in the diet [21]. African Americans report higher rates of lactose intolerance than non-Hispanic Whites [22], and our findings of disproportionally low dairy intake among non-Hispanic Black children suggest that dairy-avoidant dietary behaviors may begin in early childhood. WIC nutrition education can play a key role in closing the nutrient intake gap. For example, promoting yogurt consumption as a milk-alternative to lactose-sensitive individuals may be one strategy to increase total dairy intake among non-Hispanic Black children, and improve related nutrient disparities. Yogurt contains less lactose per serving of milk and is often tolerated by lactose-sensitive individuals [23], and yogurt consumption is associated with improved potassium and calcium status [24]. It may also be important to include dairy alternatives in the dairy category of the WIC food package, such as fortified non-dairy yogurt and non-dairy cheese. 

Among U.S. children participating in WIC, we found Hispanic children to have greater intake of both fiber and potassium compared to non-Hispanic White children. We also observed greater legume intake among Hispanic children, which may be related to the higher fiber intake. The greater legume intake among Hispanic children in this nationally representative sample is consistent with findings from regional studies of adult women participating in WIC [25], suggesting that Hispanic women and children have similar dietary behaviors that involve greater legume intake compared to their respective non-Hispanic White counterparts. For potassium intake, it is unclear which foods are driving the higher potassium intake among Hispanic children, though it is possible that this is also related to dairy consumption, as milk is a key source of potassium [26]. It is also important to consider that while the observed differences in potassium intake are statistically significant, the 135 mg difference between non-Hispanic White and Hispanic children may have limited clinical significance. 

The Dietary Guidelines for Americans (DGA) recommend limiting intake of sodium [21]. We found that average sodium intake of children participating in WIC in all racial/ethnic groups exceeded recommendations. This is consistent with trends in the U.S. adult population, with 90% of adults consuming too much sodium [27]. Despite high sodium intake among all groups, we found sodium intake among non-Hispanic Black children to be significantly greater than non-Hispanic White children. This diet-related disparity has important implications for other health disparities, given that the non-Hispanic black population has a greater propensity to salt sensitivity [28], and is overburdened with hypertension [29]. In addition, it is only in the non-Hispanic Black population that the link between sodium intake and blood pressure varies with energy intake [30], suggesting that dietary intervention may be useful in addressing both nutritional health status and blood pressure in this population. It is important to note that in adults, sodium intake of non-Hispanic Whites is greater than that of non-Hispanic Blacks [31]. Therefore, our finding of a higher sodium intake among non-Hispanic Black children relative to non-Hispanic White children reflects a different trend in the WIC-participating youth studied here compared to the published literature concerning U.S. adults. Further research is needed to better understand the relationship between WIC participation status and sodium intake among youth. 

### Strengths and Limitations

While we were able to identify associations between nutrient/food intake and race/ethnicity among children participating in WIC, NHANES data are cross-sectional and preclude causal inferences due to lack of temporality. Although 24-hour recalls are frequently used in dietary assessment, there are known limitations. Retrospective, self-reported data may be limited by recall bias (including guardian’s recall of the child’s dietary intake) or social desirability bias [32]. The results reported here represent population mean dietary intake on a given day, and therefore are not an estimation of long-term usual intake [33]. However, reporting population dietary intake from the first day of NHANES recall data promotes comparability between our findings and other studies using NHANES data to evaluate WIC participant dietary intake [7,8]. NHANES dietary data and associated dietary sample weights are commonly used to describe food and nutrient intakes on a given day, and they are also useful for descriptive and analytical epidemiologic purposes [14]. 

The findings reported here compare nutrient and food group intake among racial/ethnic groups in order to study prevalence of diet-related disparities in children participating in the WIC program. Further research statistically comparing intake estimates for each racial/ethnic group to dietary guidelines is important for influencing further recommendations for the WIC food package. In addition, we studied all foods and beverages consumed; future research should investigate the contribution of WIC-eligible foods to children’s dietary intake by race/ethnicity. Lastly, the nutrient intakes were reported for children 1–4 years of age, and food group intake analysis was performed for children 2–4 years of age, so direct comparisons cannot be made between the nutrient and food group data presented here. However, this study design enhanced comparability between our results and the age groups associated with the priority nutrients/food groups in the NASEM report [9], which served as the basis for variable selection.

## 5. Conclusions

In this study, we highlighted differences in nutrient status of children participating in WIC across racial/ethnic groups. We also analyzed disparities in food group intake to determine differences in dietary intake that may be contributing to the observed nutrient intake disparities. Compared to non-Hispanic White children participating in WIC, Hispanic children had diets with better nutrient distribution and lower dietary energy density, while non-Hispanic Black children had diets with poorer nutrient intake. Specifically, Hispanic children had lower dietary energy density, higher potassium and fiber intake, and consumed more legumes compared to non-Hispanic White children. Non-Hispanic Black children had lower calcium and vitamin D intake, higher sodium intake, and lower total dairy intake compared to non-Hispanic White children, though non-Hispanic Black children did have lower saturated fat intake. The nutrients presented here represent inadequacies and excesses linked to adverse health consequences relevant to children participating in WIC, as well as food groups priorities for Food Package IV [9], and therefore can inform future food package revisions. Further research should be done to understand how changes to the WIC food package can be used in conjunction with nutrition education, food environment intervention, and equity-oriented strategies to neutralize disparities in dietary intake and promote nutritional health of all children participating in WIC.

## Figures and Tables

**Table 1:** Sociodemographic characteristics by Special Supplemental Nutrition Program for Women, Infants, and Children (WIC) participation status for U.S. children in the NHANES 2011–2014.

**Children 1–4 Years of Age (*N* = 1072)**					
**Characteristic**	**WIC Participant**		**Nonparticipant ^a^**		***p*-Value**
	***n* = 727**		***n* = 345**		
	***n***	**weighted %**	***n***	**weighted %**	
**Sex**					0.589
Male	354	48.2%	177	50.9%	
Female	373	51.8%	168	49.1%	
**Race/Ethnicity**					<0.001
non-Hispanic White	110	30.9%	86	50.2%	
non-Hispanic Black	242	20.8%	113	17.7%	
Hispanic	305	40.3%	102	24.4%	
Other/Mixed	70	7.9%	44	7.7%	
**Income** ^b^					<0.001
PIR < 130%	539	82.7%	260	67.6%	
PIR 130%–185%	110	17.3%	85	32.4%	
**Household Size (Persons)**					0.126
2	28	2.6%	16	4.2%	
3	116	16.8%	74	21.5%	
4	168	25.2%	88	30%	
5	186	25.2%	78	24.8%	
6	97	13.5%	47	8.9%	
7	132	16.8%	42	10.6%	
**Age (Years)**					<0.001
1	218	29.2%	50	11.8%	
2	236	27.6%	101	26.1%	
3	138	21.9%	92	30.9%	
4	135	21.3%	102	31.2%	
**Children 2–4 Years of Age (*N* = 804)**					
**Characteristic**	**WIC Participant** ***n* = 509**		**Nonparticipant ^a^** ***n* = 295**		***p*-Value**
	
	***n***	**weighted %**	***n***	**weighted %**	
**Sex**					0.516
Male	248	48.2%	153	51.8%	
Female	261	51.8%	142	48.2%	
**Race/Ethnicity**					<0.001
non-Hispanic White	73	29.2%	73	50.9%	
non-Hispanic Black	173	21.7%	100	18.2%	
Hispanic	209	40.3%	89	24.7%	
Other/Mixed	54	8.7%	33	6.3%	
**Income** ^b^					<0.001
PIR < 130%	383	83.1%	223	67.8%	
PIR 130%–185%	78	16.9%	72	32.2%	
**Household Size (Persons)**					0.062
2	19	2.4%	13	4.2%	
3	76	14.6%	65	22.7%	
4	116	24.7%	75	29.0%	
5	137	26.3%	67	24.8%	
6	61	13.7%	39	8.5%	
7	100	18.3%	36	10.8%	
**Age (Years)**					0.172
2	236	39.0%	101	29.6%	
3	138	31.0%	92	35.0%	
4	135	30.0%	102	35.4%	

Sociodemographic characteristics of children living in households income-eligible for WIC during NHANES 2011–2014. ^a^ Nonparticipant was defined as children who were living in households that were income-eligible for WIC at the time of NHANES interview, but not participating in the WIC program. ^b^ Income reported as the NHANES-provided poverty income ratio (PIR).

**Table 2:** Racial/ethnic differences in nutrient intake of WIC-participating children 1–4 years of age in the NHANES 2011–2014 (*N* = 727).

	N-H White *n* = 110	N-H Black *n* = 242		Hispanic *n* = 305		Other/Mixed *n* = 70		Recommended Amount ^b^
Nutrient Category	Mean (SE)	Mean (SE)	*p*-Value	Mean (SE)	*p*-Value	Mean (SE)	*p*-Value	
Energy Density (kcal/g)	1.9 (0.1)	1.8 (0.1)	0.056	1.6 (<0.1)	<0.001	1.8 (0.1)	0.156	--
**Nutrients to Increase ^a^**								
Fiber (g)	10.7 (0.3)	10.8 (0.5)	0.812	12.1 (0.5)	0.026	9.9 (0.5)	0.129	14/16.8–19.6 (DGA)
Potassium (mg)	1936 (63)	1889 (56)	0.491	2071 (44)	0.038	1951 (117)	0.903	3000/3800 (AI)
Calcium (mg)	992 (50)	826 (39)	0.009	1012 (32)	0.684	996 (68)	0.958	700/1000 (RDA)
Iron (mg)	10.3 (0.5)	11.3 (0.5)	0.184	11.6 (0.6)	0.151	12.0 (0.8)	0.090	7/10 (RDA)
Zinc (mg)	7.3 (0.4)	7.4 (0.3)	0.730	7.6 (0.2)	0.388	7.7 (0.4)	0.380	3/5 (RDA)
Vitamin D (mcg)	7.5 (0.6)	5.7 (0.4)	0.012	6.8 (0.3)	0.187	7.4 (0.7)	0.876	15 (RDA)
**Nutrients to Limit ^a^**								
Added sugars (tsp-eq)	9.7 (1.3)	10.0 (0.6)	0.832	11.0 (1.6)	0.477	11.2 (1.6)	0.456	<10% kcal (DGA)
Saturated fat (g)	20.2 (1.1)	17.5 (0.5)	0.016	19.1 (0.6)	0.386	18.9 (1.1)	0.384	<10% kcal (DGA)
Sodium (mg)	1906 (62)	2136 (51)	0.006	2048 (38)	0.061	1987 (63)	0.339	1500/1900 (UL)

Least-square means from linear regression models are presented with standard error (SE), representing population mean nutrient intake on a given day. Analyses are for children ages one up to fifth birthday, living in households receiving WIC benefits at the time of the NHANES interview. Non-Hispanic White children are the reference group. Dietary recommendations are included for reference, statistical comparisons were not made to recommended intake. Abbreviations: N-H White, non-Hispanic White; N-H Black, non-Hispanic Black; g, grams; mg, milligrams; mcg, micrograms; tsp-eq, teaspoon equivalents; DGA, Dietary Guidelines for Americans 2015–2020; AI, Adequate Intake level; RDA, Recommended Dietary Allowance; UL, Upper Intake Level. ^a^ Nutrients analyzed represent nutrient inadequacies and excesses linked to adverse health consequences relevant to WIC-participating children from one to five years of age [9]. ^b^ Reported for children ages 1–3/children ages 4–5. In instances where recommendations differ by sex, the value is reported as a range representing female–male. Recommendations represent daily nutrition goals derived from DGA 2015–2020. Source of goal noted in parentheses [18].

**Table 3:** Racial/ethnic differences in food group intake of WIC-participating children 2–4 years of age in the NHANES 2011–2014 (*N* = 509).

Food Group Category ^a^	N-H White *n* = 73	N-H Black *n* = 173		Hispanic *n* = 209		Other *n* = 54		Recommended Amount
	Mean (SE)	Mean (SE)	*p*-Value	Mean (SE)	*p*-Value	Mean (SE)	*p*-Value	
**Higher Priority Food Group**								**DGA^b^**
Seafood (oz-eq)	0.05 (0.07)	0.20 (0.08)	0.142	0.03 (0.02)	0.756	0.14 (0.09)	0.455	0.7
Total vegetables (c-eq)	0.72 (0.17)	0.64 (0.09)	0.706	0.58 (0.08)	0.531	0.42 (0.09)	0.103	1.5
Dark green vegetables (c-eq)	0.14 (0.12)	0.01 (0.04)	0.382	<0.01 (0.04)	0.313	<0.01 (0.05)	0.295	0.1
Red and orange vegetables (c-q)	0.26 (0.09)	0.15 (0.04)	0.263	0.22 (0.04)	0.714	0.15 (0.05)	0.175	0.4
Whole grains (oz-eq)	0.89 (0.17)	0.87 (0.15)	0.946	0.74 (0.10)	0.406	0.98 (0.26)	0.689	2.3
Nuts and seeds (oz-eq)	0.34 (0.11)	0.27 (0.12)	0.627	0.16 (0.06)	0.086	0.31 (0.09)	0.608	0.4
**Lower Priority Food Group**								
Starchy vegetables (c-eq)	0.20 (0.06)	0.29 (0.06)	0.186	0.20 (0.03)	0.958	0.16 (0.07)	0.552	0.5
Other vegetables (c-eq)	0.12 (0.03)	0.19 (0.04)	0.183	0.17 (0.03)	0.261	0.14 (0.03)	0.472	0.4
Total dairy (c-eq)	2.33 (0.22)	1.75 (0.15)	0.020	2.08 (0.16)	0.247	2.16 (0.22)	0.627	2.5
Total protein foods (oz-eq) ^c^	2.19 (0.23)	3.61 (0.29)	<0.001	2.92 (0.15)	0.021	2.64 (0.29)	0.143	3.5
Legumes computed as vegetables (c-eq)	0.05 (0.03)	0.06 (0.02)	0.759	0.16 (0.02)	0.001	0.06 (0.02)	0.724	0.07

Least-square means from linear regression models are presented with standard error (SE), representing population mean food group intake on a given day. Analyses are for children ages two up to fifth birthday, living in households receiving WIC benefits at the time of NHANES interview. Non-Hispanic White children are the reference group. Dietary recommendations are included for reference, statistical comparisons were not made to recommended intake. Abbreviations: N-H White, non-Hispanic White; N-H Black, non-Hispanic Black; DGA, Dietary Guidelines for Americans 2015–2020; tsp-eq, teaspoon equivalents; c-eq, cup equivalents; oz-eq, ounce equivalents. ^a^ Food groups selected for analysis represent priorities for Food Package IV for children 2–4 years of age, as described in the NASEM report on the WIC Food Package [9]. ^b^ DGA recommendations made on a per week basis have been converted to a per day basis in order to promote comparability between results and dietary recommendations. ^c^ Total meat, poultry, seafood, organ meats, cured meats, eggs, soy, nuts and seeds; excludes legumes.
